# Is postnatal depression a distinct subtype of major depressive disorder? An exploratory study

**DOI:** 10.1007/s00737-020-01051-x

**Published:** 2020-07-15

**Authors:** Suzanne O’ Brien, Arjun Sethi, Maria Gudbrandsen, Laura Lennuyeux-Comnene, Declan G. M. Murphy, Michael C. Craig

**Affiliations:** 1 Department of Forensic and Neurodevelopmental Sciences, Institute of Psychiatry, Psychology and Neuroscience, King’s College London, 16 De Crespigny Park, London, SE5 8AF UK; 2 Department of Forensic and Neurodevelopmental Sciences and the Sackler Institute for Translational Neurodevelopmental Sciences, Institute of Psychiatry, Psychology and Neuroscience, King’s College London, London, UK; 3 National Female Hormone Clinic, Maudsley Hospital, London, UK

**Keywords:** Postnatal depression (PND), Postpartum depression, Major depressive disorder (MDD), Hormones, Functional magnetic resonance imaging (*f*MRI), Amygdala

## Abstract

Postnatal depression (PND) has an estimated prevalence of 6.5 to 12.9%. In addition to the direct consequences for women, PND also interferes with the maternal-infant interaction, contributing to long-term cognitive and emotional impairments in exposed offspring. It is unclear how PND differs from major depressive disorder (MDD) more generally, and if PND represents a distinct subtype of depression. We explored whether women with a history of PND have specific differences in brain activation associated with sex hormone changes during the late luteal phase of the menstrual cycle, compared to parous women with either a past history of MDD outside of the postnatal period, or an absent history of MDD (‘never depressed’). Thirty mothers (history of PND (*n* = 10), history of MDD (n = 10), and ‘never depressed’ (n = 10)) underwent blood-oxygen-level-dependent (BOLD) functional magnetic resonance imaging (*f*MRI) acquisition during an emotional faces task. Amygdala activity was analysed using a region of interest (small volume correction) approach. There was a significant reduction in BOLD response to positive emotional faces in the right amygdala in women with a history of PND compared to women with a history of MDD. A similar but non-significant trend was found in the left amygdala in women with a history of PND compared to ‘never depressed’ women. Our findings support the hypothesis that women with vulnerability to PND represent a distinct subgroup of women with a differential sensitivity to changes in sex hormones. Further, albeit highly tentative, they provide a putative biomarker that could assist in detection of women at-risk to PND.

**Electronic supplementary material:**

The online version of this article (10.1007/s00737-020-01051-x) contains supplementary material, which is available to authorized users.

## Introduction

The risk of major depressive disorder (MDD) is significantly increased in women following childbirth. The need for more effective treatments is supported by findings that postnatal depression (PND) is more resistant to conventional antidepressants (Hendrick et al. 2000), and suicide remains the second leading cause of perinatal death in the UK (Stein et al. 2018). Some believe that PND represents a distinct subtype of depression. However, others suggest that PND is a useful lay term, which reduces stigma, but is less useful as a medical concept (Brockington 2004). In order to develop more effective treatments, we need to better understand the biology of the disorder, and how/if PND differs from MDD.

It has been suggested that a key trigger for PND is the abrupt decrease in sex hormones that occurs postpartum (Heidrich et al. 1994), and women developing PND have differential sensitivity to these changes. This hypothesis is supported by findings that, compared to women without any history of depression, only those with a history of PND developed depressive symptoms following pharmacological suppression of gonadal steroids (Bloch et al. 2000). However, the absence of an additional control group with previous MDD (i.e. independent of childbirth) meant that it was impossible to determine whether findings were due to a specific sensitivity to hormone changes in women with PND, as opposed to MDD more generally.

Previous functional magnetic resonance imaging (*f*MRI) studies have reported, that compared to controls, patients with MDD exhibit reduced amygdala activation when viewing happy emotional faces and increased amygdala activation when presented with negative emotional faces (see Groenewold et al. 2013) for review). Whilst other brain regions have also been linked with MDD (e.g. anterior cingulate cortex (Groenewold et al. 2013)), the amygdala has been the most reliable region to have been implicated. Preliminary studies of brain function in women during an acute episode of PND, compared to controls, have reported conflicting results with, for example a reduction in amygdala activity to negative emotional faces (Moses-Kolko et al. 2010) or negatively valenced stimuli (Silverman et al. 2007). However, previous imaging studies investigating PND are small and likely to have been confounded by variables including timing of depression onset, breastfeeding status and suppression of the hypothalamic-pituitary-adrenal axis during this period. Furthermore, there have been no studies to date that have investigated whether ‘at risk’ women with a history of early-onset PND (i.e. within 8 weeks of birth (Forty et al. 2006)) have a well-defined neural response to changes in sex hormones, consistent with the concept of a distinct diagnostic nosology.

Therefore, we explored whether women with a history of PND have a specific ‘neural signature’ associated with sex hormone changes associated with the late luteal phase of the menstrual cycle, compared to women with either a history of ‘non-reproductive’ MDD, or those without any history of MDD (i.e. ‘never depressed’). We focused on the late luteal phase as the abrupt drop in plasma estradiol and progesterone concentration at this time resembles, in a scaled-down way, the postnatal hormonal milieu. Previous studies suggest that cognitive biases associated with active depression are also frequently observed following remission when patients are stressed or in a dysphoric mood (Ruhe et al. 2019). We hypothesised that the late luteal phase would represent a discrete biological stressor to women with a history of PND, but not to women in the other groups and, further, that women with a history of PND would have increased amygdala activation to negative and reduced activation to positive faces compared to both other groups.

## Methods

### Participants

We recruited 30 right-handed parous women with regular menstrual cycles aged 18–45 years (comprising 10 with a history of PND, 10 with a history of MDD, and 10 ‘never depressed’ women). Participants were screened to exclude current mental illness, premenstrual syndromes (e.g. premenstrual dysphoric disorder (PMDD)), pregnancy, IQ < 80, medical conditions affecting brain function and the current use of medication (including the contraceptive pill).

A past history of PND was defined as a diagnosis within 8 weeks of childbirth (Forty et al. 2006) (and the absence of MDD at other times). Conversely, a past history of MDD was defined by diagnosis of at least one episode of MDD in the past (and the absence of an episode following childbirth).

### Study design

We employed a case-control design to analyse the effect of late luteal phase decline in sex hormones on brain function. Participants attended the Institute of Psychiatry, Psychology & Neuroscience (IoPPN) during the late luteal phase of the menstrual cycle. Menstrual cycle tracking was assisted by home ovulation testing sticks and confirmed by a blood test on the scanning day (i.e. to measure estradiol, progesterone, follicular stimulating hormone (FSH) and luteinizing hormone (LH) concentration). This approach enabled us to target the late luteal phase more precisely.

### Psychometric and questionnaire measures

Participants underwent an investigation for psychiatric disorder using the Structured Clinical Interview for DSM-IV Axis I and II Disorders. Anxiety and depression levels were measured on the day of scanning by Beck Anxiety Inventory (BAI), Beck Depression Inventory (BDI) and the State Trait Anxiety Inventory.

### Endocrine determinants

Blood was collected into tubes without anticoagulant and allowed to clot. Samples were centrifuged for 12 min at 1500*g* and serum separated within 4 h. Hormone concentrations were quantified using competitive immunoassays (estradiol and progesterone) and two-site sandwich immunoassays (LH and FSH) using electro-chemiluminescent technology (see Table S1 in the Supplementary Material that accompanies this article).

### *f*MRI paradigms

The *f*MRI paradigm employed was the emotional faces task (Ekman and Friesen 1976) in a block design (see Supplementary Material for further information on this paradigm in the context of depression (Stuhrmann et al. 2011)). Participants were presented with positive (happy), negative (angry) and neutral facial expressions in a pseudo-randomised order, from a standardised series of prototypical facial expressions. Each facial stimulus was presented for 2 s, and the participant was asked to judge the gender of the face by pressing the left button for a female face and the right button for a male face.

### MRI acquisition

Scanning was performed using a 3 Tesla MR750 GE scanner using a 12-channel head coil. Gradient echo planar images (EPI) (FOV = 240 mm, TR/TE = 2000/30, 64 × 64 matrix, voxel size = 3.75 × 3.75 × 3.3 mm, 41 slices) were obtained for each subject. For purposes of anatomical localisation, a matched T1-weighted structural MRI (FOV = 270 mm, TR/TE/TI = 7.312/3.016/400 ms, 256 × 256 × 196 matrix, slice thickness = 1.2 × 1 × 1 mm) was acquired for each participant.

### Data pre-processing

Data were pre-processed using SPM12. EPI volumes for each subject were realigned to their mean. EPI and anatomical images were co-registered using the mean EPI and T1-weighted volumes. T1-weighted volumes were segmented, with the resulting deformation fields used to normalize subjects’ EPI volumes to the MNI template. Images were spatially smoothed using a 6-mm Gaussian kernel (FWHM).

### *f*MRI data analysis

Experimental conditions (i.e. happy, angry, neutral) were entered into the first level model as regressors of interest and modelled as blocks of 16 s. Additionally, realignment parameters for the EPI volumes were added as nuisance variables. All data were high pass filtered at 128 Hz. Contrasts of interest, ‘*Happy>Neutral’* and *‘Angry>Neutral’,* were assessed for group differences in second level analysis.

Statistical maps were initially thresholded at voxel-wise *p* < 0.005. Due to the small size of the amygdala we used a small volume correction (SVC) approach using the bilateral anatomical AAL mask *p* < 0.005, *p*_FWE_ < 0.05.

We report data from the amygdala region of interest in the main text; for completeness, we also report results from whole brain analysis in the online supplementary material.

## Results

### Demographics

Groups did not differ significantly with respect to age, IQ, BAI score, BDI score, STAI scores, hormone concentration levels, number of past depressive episodes or the average menstrual cycle day at which participants were scanned (Table 1).

**Table 1:** Sample characteristics

Mean (SD)				
	Never depressed	Past PND	Past MDD	ANOVA
Age	36.2 (5.18)	35.9 (3.7)	36.00 (5.05)	*F*(2,27) = 0.01, *p* = 0.99
IQ	111.7 (7.49)	115.6 (9.68)	116.2 (8.16)	*F*(2,27) = 0.83, *p* = 0.45
BAI	5.70 (7.28)	13.77 (17.44)	8.80 (7.39)	*F*(2,26) = 1.19, *p* = 0.31
BDI	5.60 (6.995)	11.11 (8.72)	8.00 (5.53)	*F*(2,26) = 1.41, *p* = 0.26
STAI-T	32.5 (10.86)	42.33 (15.01)	34.2 (8.14)	*F*(2,26) = 1.93, *p* = 0.17
STAI-S	37.6 (9.72)	45.66 (12.96)	43.6 (9.93)	*F(*2,26*) =* 1.43*, p* = 0.26
LH	2.58(2.28)	3.4 (2.8)	4.21 (4.06)	*F*(2,26) = 0.62, *p* = 0.55
FSH	3.65(1.57)	4.76 (2.49)	4.37 (2.66)	*F*(2,26) = 0.55, *p* = 0.58
Progesterone	34(19.95)	21.9 (22.13)	22.7 (15.56)	*F*(2,26) = 1.13, *p* = 0.34
Estradiol	539.22(212.56)	343 (217.79)	327.9 (164.22)	*F*(2,26) = 3.26, *p* = 0.06
Depressive episodes	N/A	1.3 (0.48)	1.6 (0.966)	*F*(1,18) = 0.77*, p =* 0.39
Menstrual cycle day at scan	23.5 (3.06)	23.2 (3.15)	22.75 (2.49)	*F*(2,25) = 0.14, *p* = 0.86

*PND* postnatal depression, *MDD* major depressive disorder, *BAI* Beck Anxiety Inventory, *BDI* Beck Depression Inventory, *STAI-T* and *STAI-S* State-Trait Anxiety Inventory, *LH* luteinizing hormone, *FSH* follicular stimulating hormone

### *f*MRI results

#### Region of interest

We report a significant reduction in BOLD signal in women with past PND compared to past MDD whilst viewing ‘happy’ vs. ‘neutral’ faces in the right amygdala (*p*_FWE_ = 0.044, *k* = 8, *Z* = 3.26, *xyz* = 24 − 4 − 20, *d* = − 1.49), with a trend in the left amygdala (*p*_FWE_ = 0.07, *k* = 1, *Z* = 3.10, *xyz* = 26 − 2 − 22, *d* = − 1.44).

**Fig. 1:**
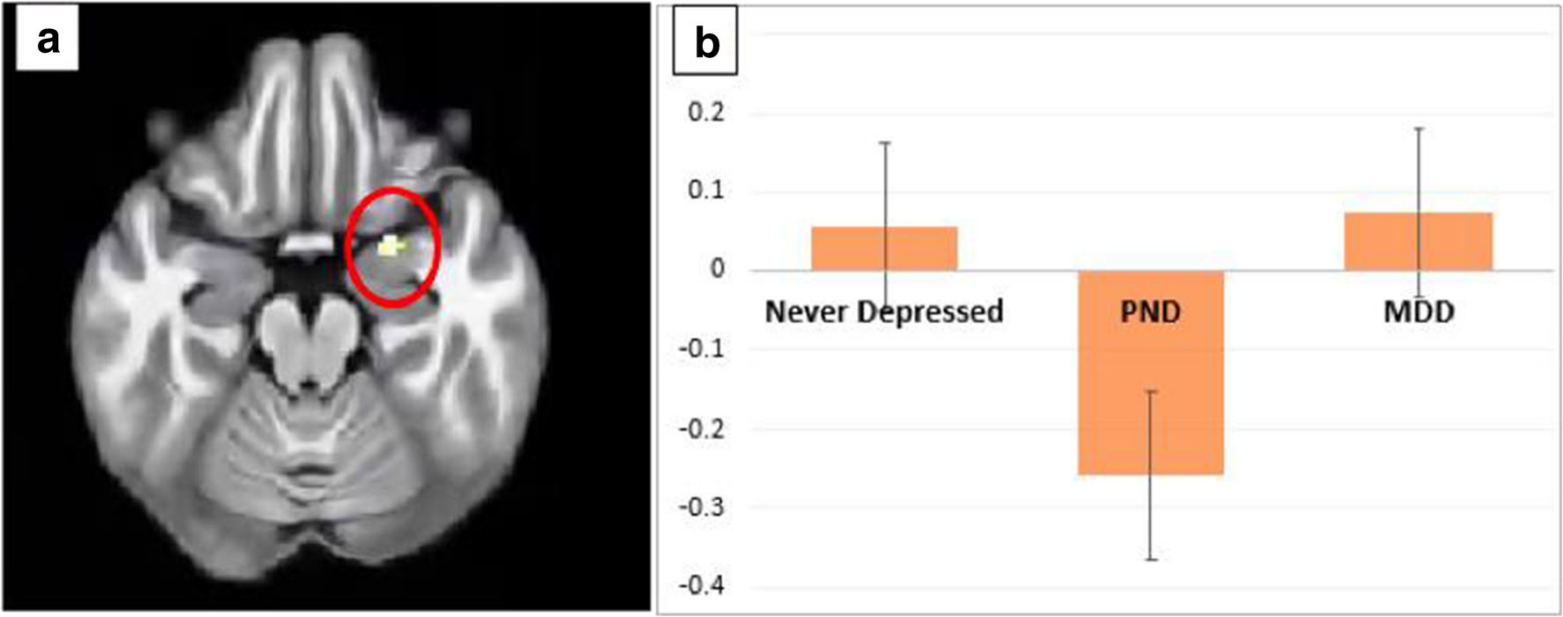
Group differences during an *f*MRI task when looking at happy faces compared to neutral faces. **a** Region of interest analysis: decrease in activation in the right amygdala in the past postnatal depression group (PND) compared to the past major depressive disorder (MDD) group (*p* = 0.044). There were no significant differences observed between the other groups. **b** Plot of mean blood oxygenation level–dependent (BOLD) signal in the right amygdala cluster with peak at coordinates at 24 4–20 (never depressed, past postnatal depression group (PND), past major depressive disorder group (MDD))

A similar but non-significant trend was also found in the left amygdala following the same contrast in women with past PND compared to ‘never depressed’ women (*p*_FWE_ = 0.08, *k* = 5, *Z* = 3.03, *xyz* = 24 − 4 − 20, *d* = 0.96) (Fig. 1)**.**

There were no significant between-group differences in BOLD response in the amygdala in contrasts of ‘angry’ vs. ‘neutral’ faces.

## Discussion

Our findings support the hypothesis that women with vulnerability to PND have a distinct neural response to positive facial emotion during the late luteal phase of the menstrual cycle. More specifically, a region of interest analysis found women with a past history of PND had significantly reduced brain activation in the right amygdala to happy faces, and a (non-significant) reduction in the left amygdala, compared to women with previous MDD, with a similar trend in the left amygdala when compared to ‘never depressed’ women. However, due to our small sample size, these results only survived with small volume correction.

The finding of reduced right amygdala activity to happy faces is consistent with a core information processing bias (i.e. attending less to positive stimuli) reported in individuals at risk of depression (Gotlib et al. 2004) and in women with PMDD (Rubinow et al. 2007). Further, in women with PMDD, increased right amygdala activity to negative stimuli (emotional words) has also been reported during the luteal phase compared to asymptomatic controls (Protopopescu et al. 2008). This study like ours was limited by its small sample size but adds support to the hypothesis that vulnerability to ‘reproductive depression’ (e.g. PMDD and PND) may be underpinned by an inherent difference in adaptation to rapid changes in neuroactive steroid concentrations. However, larger studies are needed to replicate our results and explore the relationship with angry and other negative stimuli (e.g. sad) to fluctuations in sex hormones in women with past PND, which may underpin their vulnerability to more profound changes post childbirth. Further, they suggest the need to search for putative predictive/prognostic biomarker(s) in women at-risk of PND. Larger studies are now required to replicate our findings.

## Electronic supplementary material

ESM 1 (DOCX 177 kb)
