# Supplementary material for: Is postnatal depression a distinct subtype of major depressive disorder? An exploratory study
Source: Arch Womens Ment Health. 2020 Jul 15;24(2):329–33. doi: 10.1007/s00737-020-01051-x (PMC7979595; doi:10.1007/s00737-020-01051-x)
Supplement: Supplementary file 1 — (DOCX 177 kb) [file 737_2020_1051_MOESM1_ESM.docx]

**Submission: AWMH-D-20-00022R1**

**Title: Is postnatal depression a distinct subtype of major depressive disorder? An exploratory study.**

**Supplementary material**

**Method used to ascertain menstrual phase**

Prior to the participants attending the Institute of Psychiatry, Psychology & Neuroscience (IoPPN) for their scan, each participant was asked to record the dates of their menstrual cycle for several months prior to the scan. Each participant was sent a set of home ovulation tests with instructions, and the women were requested to use one ovulation test per day from day 10 of the menstrual cycle. A researcher followed-up with the participants 2-3 times per month, (to record the menstrual cycle dates, to send reminders to start the ovulation tests and to record the dates that the women started and ovulated). Based on this data, we were confident that we could estimate a date for each participant’s scan during the late luteal phase.

Participants also gave a blood sample on the day of the scan (i.e. to measure estradiol, progesterone, follicular stimulating hormone (FSH) and luteinizing hormone (LH) concentration) to confirm they were in the late luteal phase. This approach enabled us to target the late luteal phase more precisely.

**Functional magnetic resonance imaging (*f*MRI) paradigm:**


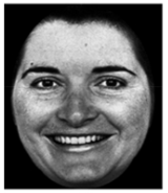

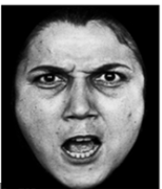

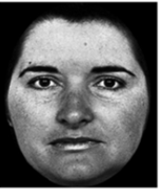


Figure S1. Faces from the standard set of Ekman & Friesen (1976). Examples of faces depicting 100% happy, angry and neutral.

The *f*MRI paradigm employed was the emotional faces task (Ekman and Friesen, 1976), which has frequently been applied to neuroimaging studies of depression (Stuhrmann et al., 2011).

Altered processing of emotional stimuli has been recognised as a core cognitive abnormality in depression, PND and PMDD (Gingnell et al., 2012; Groenewold et al., 2013; Moses-Kolko et al., 2010). Previous studies conducted in both healthy controls and women with PMDD found evidence that the emotional faces *f*MRI paradigm may be sensitive to hormonal change which may correspond to the emotional hypersensitivity frequently experienced by women postpartum (Gingnell et al., 2012; Henningsson et al., 2015). Although this has not been explored directly in women with a history of PND, it has been suggested that this may correspond to the emotional hypersensitivity frequently experienced by women postpartum (Henningsson et al., 2015).

**Hormone data**

Samples for this study were analysed on a Roche c8000 random access automated platform and blood samples were analysed on the day of collection.

Blood was collected into tubes without anticoagulant and allowed to clot. Samples were centrifuged for 12 minutes at 1500g and serum separated within 4 hours. Hormone concentrations were quantified using competitive immunoassays (estradiol and progesterone) and two-site sandwich immunoassays (LH and FSH) using electro-chemiluminescent technology (see Table S1).

**Table S1**

| Analyte | Units | QC material | Mean concentration | CV% |
| --- | --- | --- | --- | --- |
| LH | IU/L | Level 1 | 3 | 0.0 |
|  |  | Level 2 | 26 | 1.9 |
|  |  | Level 3 | 51 | 2.9 |
| FSH | IU/L | Level 1 | 6.2 | 2.4 |
|  |  | Level 2 | 25.5 | 2.4 |
|  |  | Level 3 | 58.1 | 2.3 |
| Progesterone | nmol/L | Level 1 | 2 | 25.0 |
|  |  | Level 2 | 27 | 3.7 |
|  |  | Level 3 | 87 | 3.4 |
| Estradiol | pmol/L | Level 1 | 143 | 9.8 |
|  |  | Level 2 | 836 | 3.5 |
|  |  | Level 3 | 1709 | 2.3 |

**Whole brain analysis**

In our whole brain analysis, statistical maps were initially thresholded at voxel-wise *p*<0.005 and then corrected for multiple comparisons at a family-wise error (FWE) threshold of *p*<0.05 at the cluster level.

Whole brain analyses of women with past PND, compared to ‘never depressed’ subjects, found significantly reduced BOLD signal in the raphe nuclei in the ‘happy vs. neutral’ faces contrast (*pFWE*=0.02, *k*=523, *Z*=4.23, *xyz*=0 -30 -20, *d*=1.96). There were no significant results observed in whole brain analyses between any other groups.


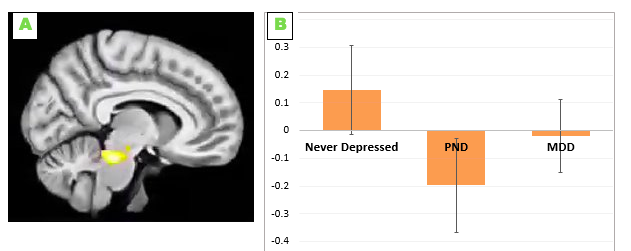


*A: Whole Brain analysis: Reduced BOLD signal in the raphe nuclei in the past PND group compared to ‘never depressed’ subjects.*

*B: Plot of mean BOLD signal in the raphe nuclei cluster with peak at coordinates at 0 -30 -20 (Never Depressed, past Postnatal Depression group [PND], past Major Depressive Disorder group [MDD])*
